# The association of carotid intima media thickness with retinol binding protein-4 and total and high molecular weight adiponectin in type 2 diabetic patients

**DOI:** 10.1186/2251-6581-11-2

**Published:** 2012-08-02

**Authors:** Masoumeh Mansouri, Ramin Heshmat, Ozra Tabatabaei-Malazy, Farshad Sharifi, Zohreh Badamchizadeh, Sudabeh Alatab, Kobra Omidfar, Hossein Fakhrzadeh, Bagher Larijani

**Affiliations:** 1 Endocrinology and Metabolism Research Center, Tehran University of Medical Sciences, P.O. Box 14395/1179, Tehran, IR, Iran

**Keywords:** Carotid intima media thickness, Adipokines, B mode ultrasonography, Enzyme linked immunosorbant assay (ELISA), Type 2 diabetes

## Abstract

**Background:**

The aim of this study was to investigate whether carotid intima media thickness (CIMT) is associated with serum level of retinol- binding protein-4 (RBP4) and total and high molecular weight (HMW) adiponectin in type 2 diabetes (T2DM) without clinical symptom of atherosclerotic disease.

**Method:**

101 type 2 diabetic patients (mean age, 53.63 ± 8.42 years) and 42 body mass index (BMI) matched control (mean age 50.1 ± 8.4) were recruited. The CIMT was assessed by using B-mode ultrasonography, while serum levels of RBP4 and total and HMW adiponectin were measured by using enzyme linked immunosorbant assay (ELISA). Linear regression analysis was performed with CIMT as dependent variable and adipokines and cardio metabolic risk factors as independent variables.

**Result:**

The CIMT was higher in diabetic group compared to control group (p <0.05). The mean concentration of RBP4 and total and HMW adiponectin did not differ between two groups.

Age (B = 0.44 P <0.05), blood pressure (B = 0.37 P = <0.05), waist circumference (B = −0.21 P <0.05) and TG (B = 0.1 P <0.05) were identified as independent predictors for CIMT in diabetic group, while RBP4 and adiponectin were not associated with CIMT neither in diabetic group nor in control group.

**Conclusion:**

In conclusion, the present study showed that serum levels of RBP4 or total and HMW adiponectin were not potential predictors of CIMT in type 2 diabetic patients who exposed to this risk factor at least for nine years.

## Introduction

The type 2 diabetes (T2DM) is one of the major risk factors of cardiovascular disease (CVD) which is in turn the leading cause of death in these patients [1].

Therefore, one of the most important issues in the management of diabetes is to predict and prevent the development of CVD [2]. The carotid intima- media thickness (CIMT) is an independent predictor of future cardiovascular events and increased CIMT was recently suggested to be significantly related to the presence and extent of abnormal myocardial perfusion. Therefore the CIMT can be used as a screening test in the diabetic patients who are at risk of coronary artery disease (CAD) [3,4].

Recent studies have shown that adipose tissues secret a number of various bioactive substances known as adipokines which might affect insulin sensitivity or vascular function [5-8]. Of these adipokines, retinol binding protein-4 (RBP4) acts as a potential determinant of insulin resistance and plays a role to induce sub clinical inflammation leading to cardiovascular diseases in T2DM [7,9]. It was reported that serum RBP4 levels were increased in individual with obesity, impaired glucose tolerance, T2DM and even gestational diabetes [10-13].

Inversely previous data showing that adiponectin has anti-inflammatory properties and suppresses various mechanisms contributing to atherogenesis [14]. According to the literature plasma adiponectin may be deregulated in disorders susceptible to atherosclerotic vascular diseases, such as diabetes mellitus [15].

Increasing adiponectin by life style change and exercise has been reported in diabetic patients but not in healthy subject [16]. Regarding the relationship between CIMT and these two adipokines a few information with conflicting results have been published. Moreover the place of these adipokines measurements as plasma markers to predict cardiovascular disease in diabetic patients is not yet clear. Therefore this study was designed to investigate the associations between CIMT and RBP4 and total and HMW adiponectin altogether in diabetic patients who did not have clinical symptom of atherosclerotic vascular disease.

## Methods

### Subjects

The study samples included a total of 101 type 2 diabetic patients (48 male and 53 female) with a mean age of (53.6 ± 8.4 year) who were diagnosed according to the world health organization criteria as two times fasting blood glucose equal or greater than 126 mg/dl or use of oral hypoglycemic agent. They were visited in a diabetes outpatient clinic in Shariati Hospital of Tehran University of Medical Sciences. The mean duration of diabetes was (8.85 ± 6.24) years. All diabetic patients were on oral anti diabetic agents and none of them were current or previous smoker except nine. The control groups were selected among healthy friends of diabetic patients who volunteered to participate in a medical and laboratory screening examination, including 25 males and 20 females, with a mean age of (50.1 ± 8.4 years). The inclusion criteria for control group included healthy subject without diabetes and known metabolic disease and did not take any medication. We collected information on lifestyle, medical history, smoking and the use of medication by using questioners (which were requested to fill out by all the participants) and subsequent interview. Both groups were within the same age interval and matched for BMI.

The diabetic cases who had clinical symptoms of large vessel diseases including chest pain, abnormal 12-lead electrocardiography, history of myocardial infarction, cerebral infarction and claudication were excluded. In addition for evaluating peripheral vascular disease (PVD) ankle brachial pressure index (ABI) was measured with a Doppler Ultrasound (France). The patients with ABI less than 0.9 and more than 1.3 were excluded [17]. Screening for microvascular complication was performed as follows. Assessment of diabetic retinopathy as no diabetic retinopathy, non proliferative retinopathy and proliferative diabetic retinopathy were performed by two trained ophthalmologist. The subjects with proliferative retinopathy were excluded. Renal dysfunction was assessed by estimated glomerular filtration rate using the modification of diet in renal disease equation (GFR-MDRD) [18]. For evaluating neuropathy, vibratory sensation was examined by placing a 128 hz diapason on the malleolar and the radius of lower extremity and upper extremity respectively. Vibratory sensation seems to be adversely affected early in the pathogenesis of diabetic peripheral poly-neuropathy [19]. The patients with vibration sensation loss were excluded.

Protocol of the study was approved by ethics committee of the Teheran University of Medical Sciences and a written consent form with the required information was signed by the participants prior to the study.

## Methods

After at least 12 hours fasting, blood samples were collected. Height, weight and resting blood pressure in a sitting position were measured by the study attending nurse.

 The BMI was measured by dividing the weight (kg) to height (m^2^). Waist circumference defined as the minimal abdominal circumference between the xiphoid process and iliac crest.

Hypertension was defined as systolic blood pressure (SBP) ≥ 140 mm/Hg or diastolic blood pressure (DBP) ≥ 90 mm Hg and or using anti-hypertensive medication in the same session. The blood was transformed immediately and kept at -80^c^ C. Fasting plasma glucose (FPG), total cholesterol and triglyceride were measured by commercially available enzymatic reagents (Pars Azmoon, Tehran, Iran) adapted to an Auto analyzer (Hitachi 902, Japan). High density lipoprotein cholesterol (HDL-C) and low density lipoprotein cholesterol (LDL-C) were assayed by using turbidometric method (Pars Azmoon, Tehran, Iran).

Insulin was measured via ELISA using Monobind kit (Denmark), with inter-assay and intra- assay coefficient variation (CV) being 6.32% and 1.9% respectively. HbA1C levels are measured by High-performance liquid chromatography (HPLC). Homeostasis model assessment (HOMA) index was applied for the determination of the insulin resistant according to the following equation: HOMA index = fasting glucose (mg/dl) x fasting insulin (μU/ml) / 405.

Serum RBP4 was measured using ELISA kit (AdipoGen, Seoul, Korea). The total and HMW adiponectin were also assessed using ELISA kit (Millipore, USA). Three samples of known concentration (low, moderate and high) were tested for both RBP4_,_ the total and HMW adiponectin in eight independent analytical runs to assess precision within and between assays. The measured intra-assay (CV) for low, moderate and high range of RBP4 were 3.5%, 2.0% and 3.7%, respectively, and the inter-assay were 7.1%, 8.2% and 7.4%, respectively. The measured intra-assay CV for total adiponectin was 7.5% and the inter-assay was 6.25%. The measured intra-assay CV for low, moderate and high range of HMW adiponectin were 1.02%, 2.68% and 2.62%, respectively, and the inter-assay were 4.1%, 7.2% and 4.03%, respectively.

CIMT was measured by B- mode ultrasonography which was equipped with a 13 MHZ linear transducer and performed by 2 expert radiologists for all participants. Patients were in the supine position with a little hyper extension and rotation of the neck to the opposite side. The measurements were acquired from three segments including common carotid artery (CCA), carotid bifurcation and internal carotid artery on both left and right sides posteriorly and anteriorly at the end of diastolic phase. The CIMT was measured at the far wall on each carotid segment on both sides as the distance between the interface of the lumen and intima, and the interface between the media and adventitia. The average value of CIMT measurements of twelve locations was expressed as mean CIMT.

### Statistics analysis

Normality assumption of variables was defined by Kolmogorov-Smirnov test and then the comparison between groups mean differences was performed by student’s t test. Normal distributed data are expressed as the mean ± SD. Because of skewed distribution, adiponectin and CIMT concentration were logarithmically transformed. The relationship between CIMT and adipokines was tested by using the spearman correlation and regression models. Univariate analysis of baseline clinical, metabolic markers, and serum adipokines including age, sex, smoking, BMI, waist circumference, blood pressure, fasting glucose, triglyceride, total cholesterol, HDL and LDL cholesterol, serum creatinin. RBP4 and total and HMW adiponectin and ABI was performed separately in diabetic and control groups. Subsequently, risk factors with a significant P-value (<0.05) were included in a linear multiple regression model to determine independent predictors of CIMT. The SPSS 11.5 statistical software package (SPSS Inc. Chicago) was applied for all calculations. A 5% or lower p-value is considered statistically significant

## Results

### Clinical characteristics of patients

The clinical and metabolic features of variables belonging to diabetic patients and controls and also information about the number of subjects taking any medication were shown in Table 1.

**Table 1:** Clinical characteristics of diabetic group and control group

**Variables**	**Cases**	**Controls**	**P**	**Age adjusted**
sex (m/f)	101 (48/53)	42 (19/23)		
Age (years)	53.6 ± 8.4	50.1 ± 8.4	<0.05^*^	
BMI (kg/m2)	27.7 ± 4.1	28.7 ± 4.6	0.37	0.26
Waist circumference (cm)	94.0 ± 10.8	93.5 ± 10.3	0.81	0.89
Duration of Diabetes (years)	8.8 ± 6.2	-	-	
SBP (mm Hg)	132.9 ± 17.3	129.1 ± 13.6	0.19	0.39
DBP (mm Hg)	77.0 ± 9.7	78.7 ± 11.2	<0.05^*^	0.46
FPG (mg/dl)	165.1 ± 62.2	97.8 ± 10.5	0.001^*^	0.001^*^
HbA1C	7.9 ± 1.7	5.3 ± 0.6	0.001^*^	0.001^*^
Fasting Insulin	8.6 ± 6.6	8.5 ± 4.9	0.95	0.76
HOMA-IR	3.5 ± 2.7	2.1 ± 1.2	0.001^*^	0.001^*^
Triglyceride	195.3 ± 108.1	171.8 ± 84	0.19	0.12
Total cholesterol	174.6 ± 39.4	201.4 ± 39.5	0.001*	0.001*
HDL cholesterol	40.7 ± 8.9	45.9 ± 11.7	0.01^*^	0.01^*^
LDL cholesterol	94.9 ± 23.8	113.9 ± 24.8	0.001^*^	0.001^*^
Serum creatinine (mg/dl)	1.0 ± 0.2	0.9 ± 0.2	0.45	0.29
eGFR (ml/min/1.73 m2)	77.0 ±14.2	77.3 ± 11.2	0.9	0.25
Current smoking, (n) (%)	9(8.6)	1(2.6)		
Adipokines				
RBP4 (µg/ml)	71.9 ± 35.6	80.7 ± 31.6	0.15	0.21
Total^a^ adiponectin (µg/ml)	6.6 ± 3.4	6.62 ± 2.8	0.82	0.72
HMW^a^ adiponectin (µg/ml)	3.7 ± 1.9	3.50 ± 1.7	0.90	
CIMT (mm)	0.8 ± 0.2	0.66 ± 0.2	0.001^*^	0.04^*^
ABI	1.1 ± 0.1	1.2 ± 0.1	0.015	0.97
Medication				
Metformin (n) (%)	78 (77.2)	-		
Glybenclamide (n) (%)	78 (77.2)	-		
Statin (n) (%)	43 (42.5)	-		
Aspirin (n) (%)	46 (45.5)	-		

BMI, body mass index ;SBP, systolic blood pressure; DBP, diastolic blood pressure; FPG, fasting plasma glucose; HOMA-IR homeostasis model assessment of insulin resistance; HDL, high-density lipoprotein; LDL, low-density lipoprotein; eGFR, estimated glomerular filtration rate ; RBP4, retinol-binding protein 4; HMW, high molecular weight adiponectin; C IMT, carotid intima-media thickness. ABI, ankle brachial pressure index.

According to the t test the diabetic patients were older and had significantly higher levels of serum FBS, HbA1C and HOMA index as expected, whereas serum total cholesterol, HDL and LDL cholesterol were significantly higher in control group.

The mean concentration of RBP4 and total and HMW adiponectin did not differ between two groups. CIMT was significantly higher in diabetic group [0.76 ± 0.16 vs. 0.66 ± 0.16 p = 0.001] while ABI was significantly higher in control group (1.11 ± 0.10 vs. 1. 16 ± 0.11 p = 0.015). To reduce the effect of age which were significantly higher in diabetic group, age adjusted analysis were performed but it did not modify our significant findings except for ABI which did not remain significant anymore between the two groups (Table 1).

### The association of CIMT with RBP4 and total and HMW adiponectin

**Figure 1:**
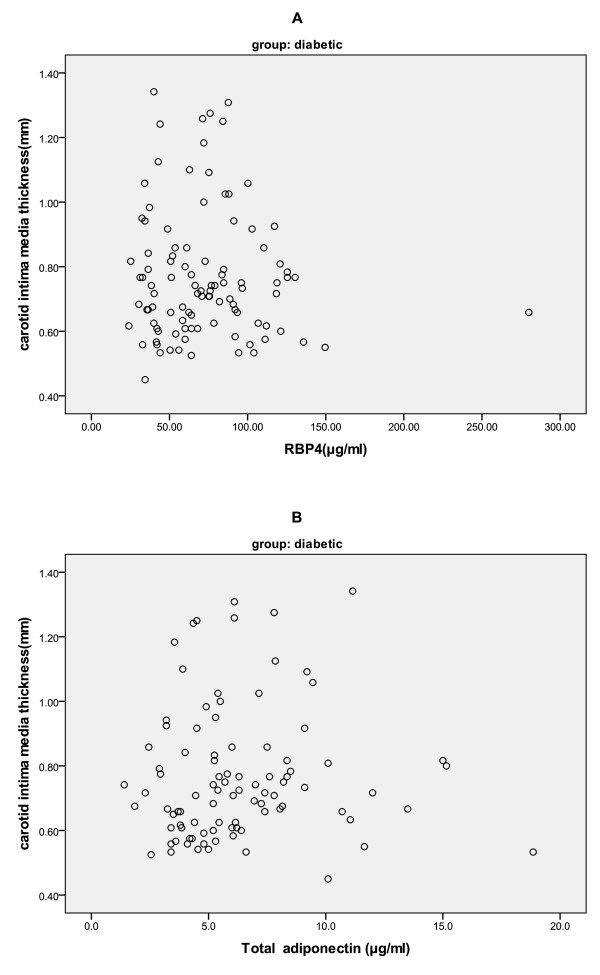
Scatterplot of Carotid intima media thickness and serum concentration of RBP4 (A) and total adiponectin (B).

No significant relationship was observed between CIMT and RBP4 (r = 0.008 p >0.05) or total (r = 0.125 p >0.05) and HMW adiponectin (r = 0.109 p >0.05) by using spearman correlation (Figure 1). Linear regression analysis was also performed with CIMT as dependent variable and adipokines and cardio metabolic risk factors as independent variables.

As shown in Table 2, age, waist circumference, TG were identified as independent predictors for CIMT while RBP4 and adiponectin were not potential predictor of CIMT neither in diabetic group nor in control group. Even though ABI tended to have negative association with CIMT, but it still did not reach to significant level. Importantly in multivariate model, age, blood pressure and triglyceride were the main predictors of CIMT.

**Table 2:** The relationship between CIMT and RBP4, total and HMW adiponectin and other cardio metabolic risk factors as independent variable

	**Diabetic group**				**Control group**	
	**Univariate**		**Multivariate**		**Univariate**	
**Variable**	**Standard B coefficient**	**P value**	**Standard B coefficient**	**P value**	**Standard B coefficient**	**P value**
Age	0.44	0.01	0.328	0.001	0.35	0.01
Sex	−0.11	0.23	-	-	−0.21	0.18
Smoking	0.07	0.43	-	-	0.15	0.82
Blood pressure	0.37	0.01	0.246	0.006	0.16	0.33
Waist circumference	−0.21	0.03	−0.219	0.010	0.002	0.981
BMI	−0.13	0.18	-	-	−0.16	0.31
FPG	−0.07	0.45	-	-	−0.04	0.48
Creatinine	0.11	0.23	-	-	0.04	0.76
Triglyceride	0.12	0.05	0.243	0.007	−0.02	0.82
Total -C	0.15	0.18	-	-	0.05	0.71
HDL-C	−0.01	0.87	-	-	0.09	0.54
LDL-C	0.14	0.1	-	-	−0.10	0.53
RBP4	−0.06	0.54	-	-	−0.2	0.20
Total Adiponectin	−0.07	0.58	-	-	−0.06	0.88
HMW Adiponectin	0.07	0.53	-	-	−0.08	0.07
ABI	−0.1	0.07	-	-	−0.008	0.96

BMI, body mass index; FPG, fasting plasma glucose; HDL, high-density lipoprotein; LDL, low-density lipoprotein; RBP4, retinol-binding protein 4; HMW, high molecular weight adiponectin; ABI,ankle brachial pressure index.

## Discussion

In this study we evaluated the association of CIMT, an established marker of subclinical atherosclerosis, with RBP4 and total and HMW adiponectin in type 2 diabetic patients without clinical evidence of atherosclerotic vascular disease. We found that the CIMT in diabetic group was significantly higher compared to non-diabetic group. This finding is in agreement with previous study that reported the chronic hyperglycemia in diabetic patients could induce athrogenesis by increasing oxidative stress and decreased nitric oxide bioavailability [9]. It is well known that the risk of atherosclerosis is much higher in diabetic patients and suggested that, in response to diabetes the endogenous defense of the vascular endothelium begins to break down [20]. The main finding of our study is that, the CIMT did not show a significant relationship with total or HMW adiponectin in a univariate regression analysis. Our result is inconsistent with some previous studies reported an inverse correlation between adiponectin levels and CIMT in non-diabetic subjects including middle aged healthy women [21], obese children [22] and healthy middle age males [23]. A negative correlation has also been shown between HMW adiponectin level and CIMT in obese and normal weight adolescents [24]. However the lack of correlation between CIMT and adiponectin has also been shown in healthy males [25] and middle–aged non diabetic women [26].

Taking together, it seems that the reported association of CIMT with total and HMW adiponectin is stronger in normal adults and obese children than in diabetic patients.

Regarding to RBP4, in multivariable analysis, adjusted for known risk factors which affect on CIMT, no independent association between CIMT and this adipokine was found. In the literature, there are several controversial reports regarding association between CIMT and RBP4. For example, Ingelsson et al. [27] assessed the relation between RBP4 and CIMT in healthy elderly subjects and found an inverse correlation of RBP4 with intima- media and plaque echogenicity. However, more recent data do not confirm those findings in T2DM. In a study performed in type 2 diabetic patients by Takebayashi and his colleagues [28], the CIMT showed no association with RBP4. Lack of correlation between CIMT and RBP4 has also been reported in a recent published article performed on newly diagnosed un-treated type 2 diabetes [29]. The possible reasons for not finding significant association between CIMT and adipokines in our study are explained below. Measurements of CIMT could reflect the vascular structural changes including intimal lesion and medial hypertrophy [30] due to past long term exposure to risk factors [31]. While it could have been the biological effect of these adipokines precedes the structural changes determined by CIMT. As some evidences showed that these adipokines may act as determinant of the early functional change of the vascular system [8]. Endothelial dysfunction occurs during the early stages of atherosclerosis and is responsible for the pathophysiological changes leading to subclinical atherosclerosis determined by CIMT [32]. Endothelial dysfunction resulted from decreased production or availability of NO which is the key factor playing an essential role in the regulation of vascular function [33,34].

The result of the study performed by Beauloye et al. [8] showed that adiponectin level were independently associated with increased IMT and they suggest that adiponectin may play an early role in the pathophysiology of atherosclerosis. Established by in vitro studies, adiponectin serves to protect against the onset of endothelial dysfunction by promoting NO generation [35]. Conversely RBP4 may affect endothelial function directly through inhibition of insulin mediated pathway for nitric oxide production in endothelial cells [29,36].

Moreover some studies showed, adiponectin inhibits expression of adhesion molecules which induces the progression of atherosclerosis [37,38]. In contrast a positive correlation between RBP4 and several soluble adhesion molecules are reported [28]. In addition some studies suggested that RBP4 might be responsible for up regulation of endothelial adhesion molecules and development of vascular complication [30,39,40].

Based on these evidences it seems these adipokines are more related to the markers which induce early stages of vascular changes than established structural changes reflected by CIMT. While the mean duration of diabetes in this study population was nearly nine years (Table 1) and it is not known for how long the patients have been exposed to this major risk factor prior to the confirmed diabetes diagnosis. Therefore, further studies on young adult who are prone or newly exposed to diabetes, are needed to determine whether the serum levels of these adipokines could act as plasma markers to predict vascular thickness.

Another reason for not finding this association is that, many diabetic patients in this study were taking a range of medication that might affect the CIMT measurements, serum level of adipokines and on the relationship between them.

In our study, CIMT showed a significant and positive correlation with age and systolic blood pressure and triglyceride but it did not show a significant relation with any of metabolic markers including fasting glucose, insulin and lipid markers.

The lack of association between CIMT and fasting glucose, insulin and lipid markers has been reported by previous investigators [15,41] and could be explained at least partially by taking into account the role of anti-hyperglycemic and anti-hyperlipidemic medications consumed by diabetic patients.

In conclusion, the present study showed that serum levels of RBP4 or total and HMW adiponectin were not potential predictors of CIMT, which reflects vascular structural changes, in type 2 diabetic patients who exposed to this risk factor at least for nine years.

## Competing interests

Authors are confident that they are not affected by conflicts of interests.

## Authors’ contributions

MM: First author, proposal writer, study researcher, interpreted the results, added data and their interpretation, wrote the paper and revised the paper. RH: analyzed and interpretation the data. OT-M: co- study designer, study researcher. FS: Interpretation the data. ZB: Collecting data and interview with the patients. SA: English editor. KO: First corresponding author, study designer, supervisor of conduction of the study and writing the paper. HF: Second corresponding author, supervisor of collecting data. BL: Co-study designer. All authors have contributed to, seen and approved the manuscript.
